# Effect of Sodium Butyrate on *LHX1* mRNA Expression as a Transcription Factor of HDAC8 in Human Colorectal Cancer Cell Lines

**Published:** 2019

**Authors:** Mahsa Ghiaghi, Flora Forouzesh, Hamzeh Rahimi

**Affiliations:** 1. Department of Genetics, Faculty of Advanced Science and Technology, Tehran Medical Sciences, Islamic Azad University, Tehran, Iran; 2. Department of Molecular Medicine, Pasteur Institute of Iran, Tehran, Iran

**Keywords:** Colorectal cancer, HCT-116 cells, Histone deacetylase inhibitors, Humans, Transcription factors

## Abstract

**Background:**

LHX1 is an important transcription factor for the *HDAC8* gene. The aim of this study was to investigate the effect of Sodium Butyrate (SB), as a histone deacetylase inhibitor, on the expression of *LHX1* gene in colorectal cancer cell lines.

**Methods:**

HT-29 and HCT-116 cell lines were treated with 6.25 to 200 *mM* concentrations of SB at 24, 48, and 72 *hr*. The cytotoxicity effect on cell viability was evaluated by MTT assay. The 50% Inhibiting Concentration (IC_50_) was determined graphically. Quantitative real-time PCR was performed to investigate the *LHX1* mRNA expression level.

**Results:**

Our study revealed that SB inhibited the proliferation of these cell lines in a concentration and time-dependent manner. The IC_50_ values for HT-29 cell line were 65, 18.6, and 9.2 *mM* after 24, 48, and 72 *hr* of treatment, respectively. The IC_50_ values for HCT-116 cell line were 35.5, 9.6, and 10 *mM* after 24, 48, and 72 *hr* of treatment, respectively. Furthermore, real-time PCR findings demonstrated that the *LHX1* mRNA expression in treated HT-29 cell line significantly increased in comparison with untreated cells (p<0.05). However, in treated HCT-116 cell line, SB led to a significant decrease in the level of *LHX1* mRNA (p<0.05), as compared to untreated cells.

**Conclusion:**

In this study, different effects of SB on *LHX1* mRNA expression level were revealed in two distinct human colorectal cancer cell lines.

## Introduction

Colorectal cancer is one of the most common cancers in the world including 9% of all cancers 1. This cancer is the second common cancer and the fourth cause of death due to cancer globally 2. Dysregulation in the epigenetic mechanisms, including histone acetylation, is one of the main factors contributing to the colorectal cancer 3–5. Acetylation, a process in which the chromatin structure and gene expression 6 are modified, is controlled by two types of enzymes, Histone Acetylases (HAT) and Histone Deacetylases (HDACs) 7. The change in acetylation status in cancer cells such as prostate 8, colon 9, and gastric 10 cancers has been linked to the increased expression of certain HDAC in indefinite patterns.

HDACs directly interact with transcription factors and can regulate the expression of a large number of genes 11. LHX1 (LIM Homeobox1) protein is one of the transcription factors involved in the transcription of *HDAC8* gene 12. Moreover, it has different functions including regulation of cell fate, cellular skeleton organization, and tumor formation 13–16. The *LHX1* expression has been reported in human cancers such as ovarian cancer, kidney carcinoma, leukemia cells, and epithelial cells 17.

Histone Deacetylase Inhibitors (HDACi) can change the balance between HAT and HDAC, and also lead to the acetylation of histone and non-histone proteins that induce transcription and related molecular effects 18. Some processes involved in the inhibition of HDAC are apoptosis, necrosis, growth inhibition, and differentiation 19–21. One of the HDACi is Sodium Butyrate (SB) 22,23. The produced butyrate in the colon may inhibit the development of colon cancer and protect against colon cancer 24,25. One of the functions of butyrate is its anti-inflammatory effect that plays a crucial role in inhibiting the histone deacetylase 26. In addition, SB influences the gene expression through binding to the transcription factors. Epigenetic regulation orchestrates various physiological procedures, comprising transcription, replication, and repair from developmental to differentiated stages and emerges with a pivotal role in the process of tumorigenesis 27–29. The understanding of these mechanisms might contribute to the optimization of prognostic and diagnostic systems, as well as the generation of novel and targeted therapeutic approaches. In the present study, the effect of SB on *LHX1* mRNA expression, as a transcription factor of the *HDAC8* gene, in HT-29 and HCT116 human colorectal cell lines was investigated. It is expected that the expression of *LHX1* in treated cells would be decreased, in comparison with untreated cells*.* Our results showed that in HCT-116 cells, the expression of *LHX1* was decreased; however, in HT-29 cells this expression level was increased, compared with untreated cells. One of the explanations for this may be the different tissue origin of these two cell lines given the fact that HT-29 is adenocarcinoma and HCT-116 is carcinoma. Furthermore, these cell lines represent a wide range of cancer characteristics; HCT-116 has a wild-type p53 response while being deficient in mismatch repair, whereas the HT-29 is p53 deficient and an unstable cell line 30. Molecular mechanisms may affect the underlying function in each cell line.

## Materials and Methods

### Cell culture

HT-29 and HCT116 human colorectal cell lines were purchased from Pasteur Institute of Iran (Tehran, Iran). HT-29 and HCT116 cells were cultured in RPMI 1640 and DMEM (Dulbecco’s Modified Eagle’s Medium) (Gibco, Germany), respectively, which was supplemented with 10% heat-inactivated fetal bovine serum (FBS) (Gibco, Germany) and 1% penicillin-streptomycin (100 *IU/ml* and 100 *μg/ml*, respectively) (Dacell, Iran). Cells were incubated at 37*°C* under a humidified atmosphere of 95% air and 5% CO_2_ (*v/v*). Monolayer cells were harvested by 0.25% trypsin-EDTA (Gibco, Germany).

### SB treatment

Optimization of cell numbers in 96-well plates (Spl life sciences, Korea) was performed for 24, 48, and 72 *hr* of incubation time. A total of 50×10^3^ cells per well (The optimized cell number) were seeded in 96-well plates and incubated for 24 *hr*. SB was dissolved in sterile water with a 1 molar concentration of stock solution for *in vitro* studies, which was further diluted to the working concentration (6.25 to 200 *mM*) in culture media. All cell lines were then treated with SB at the concentrations ranging from 6.25 to 200 *mM* for 24, 48, and 72 *hr*. Untreated cells (0 *Mm*) and cells treated with dimethyl sulfoxide (DMSO) 20% were considered as negative and positive controls, respectively.

### Cytotoxicity assay

The cytotoxic effect of SB (Biobasic, Canada Inc.) in HT-29 and HCT-116 colorectal cell lines was determined using the 3-(4,5-dimethylthiazol-2-yl)-2,5-diphenyltetrazolium bromide (MTT) assay (Sigma, USA) and was compared with the untreated cells (0 *Mm*) as a control group. Briefly, 100 *μl* of the MTT stock solution (5 *mg/ml* in PBS) was added to each well to attain a final concentration of 0.5 *mg/ml* in RPMI-1640 without phenol red culture (Biosera, France). After 4 *hr* of incubation, the supernatants were aspirated; the formazan crystals in each well were dissolved in 50 *μl* DMSO and the absorbance was measured at 546 *nm* using an ELISA reader (Garni Medical Eng. Co., Tehran, Iran). Each SB concentration was assayed in separated wells and each experiment was repeated at least 3 times. Cell viabilities were calculated using the following formula:

$$\text{Cell}\;\text{viability}\;\text{rate}\;(\%)=({\text{OD}}_{546}\;\text{of}\;\text{treated}\;\text{cells}/{\text{OD}}_{546}\;\text{of}\;\text{control}\;\text{cells})\times 100\%.$$

Afterwards, the half-maximal growth inhibitory concentration (IC_50_) values were estimated from dose response curves by applying linear regression analysis via the JavaScript version of PolySolve (07.20.2013) software.

### RNA extraction and cDNA synthesis

A total of 3×10^6^ HT-29 and HCT-116 human colorectal cells were seeded in 6-well plates (Spl life sciences, Korea) in 2 *ml* of RPMI-1640 and DMEM medium supplemented with 2% FBS, respectively, and were treated with different concentrations of SB (6.25 to 200 *mM*) for 24 and 48 *hr*. After the end of incubation time, total cellular RNA was extracted from the cancer cells treated with SB and untreated cells using RNX-Plus Solution (Sinaclon, Iran). The quality and quantity of extracted RNA were measured with agarose gel electrophoresis and a spectrophotometer (Eppendorf, Germany). Complementary DNA (cDNA) was synthesized with 2000 *ng* total RNA using a cDNA synthesis kit (Yektatajhiz, Iran) according to the manufacturer's protocol.

### Quantitative real-time PCR (qRT-PCR)

The qRT-PCR analysis was carried out for *LHX1* gene using RQ-PCR SYBR Green I system Light Cycler 96 (Roche Diagnostics, Germany). The *GAPDH* (Housekeeping gene) was used as an internal control. Reactions were prepared in duplicate using 2X SYBR Green Supermix (Pishgam, Iran) according to manufacturer’s instructions to a final volume of 20 *μl*. The following conditions were used: 95*°C* for 15 *min*, followed by 40 cycles of denaturation at 95*°C* for 15 *s*, annealing, and extension at 60*°C* for 60*s*. Quality of PCR products was evaluated by generating a melting curve, which was also used to verify the absence of PCR artifacts (Primer-dimers) or nonspecific PCR products. Variations in relative gene expressions between treated cells and control group (Untreated cells) cDNA samples were identified with Relative Expression Software Tool 9 (REST 9, Qiagen) using the 2^−ΔΔCT^ method. The primers (10 *pmol*) are listed in table 1.

**Table 1.** Primer sequences used in quantitative polymerase chain reaction (qRT-PCR)

**Name**	**Forward primer sequence (5′–3′)**	**Reverse primer sequence (5′–3′)**	**Accession number**
***GAPDH***	GAAGGTGAAGGTCGGAGTC	GAAGATGGTGATGGGATTTC	NM_001289745.2
***LHX1***	TCTCCAGGGAAGGCAAACT	CGAAACACCGGAAGAAGTC	NM_005568.4

### Data analysis

Ct values were adjusted, taking into account primer efficiencies for each gene when calculating 2^−ΔΔCT^ values. Expression data for each target gene was also normalized to the housekeeping gene (*GAPDH*) and fold change calculations were made based on Schmittgen and Livak’s method by using REST 9 and LinRegPCR softwares. The level of statistical significance was set at p<0.05.

## Results

### The effect of SB on the cell viability of HT-29 and HCT-116 human colorectal cancer cell lines

**Figure 1.**
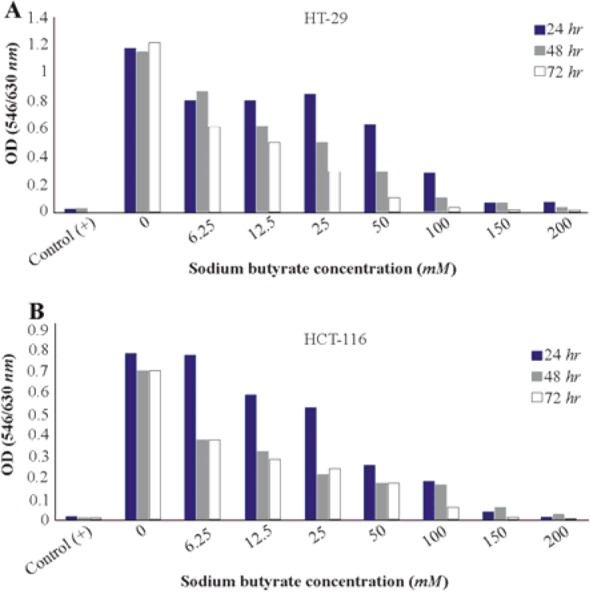
Cell viability in cancer cells treated with sodium butyrate (SB). A) HT-29 colorectal cell line was treated with 6.25 to 200 *mM* concentrations of SB at 37*°C* for 24, 48, and 72 *hr* of incubation. B) HCT-116 colorectal cell line was treated with 6.25 to 200 *mM* concentrations of SB at 37*°C* for 24, 48, and 72 *hr* of incubation. Cell viabilities were evaluated using MTT assay and calculated as a ratio of the control. Control (+): cells treated with dimethyl sulfoxide (DMSO) 20% and untreated cells (0 *mM*) as negative control. All experiments were performed in triplicate.

To investigate the role of HDAC on the proliferation of colorectal cancer cells, HT-29, and HCT-116 human colorectal cell lines were treated with various concentrations of SB (From 6.25 to 200 *mM*) for 24, 48, and 72 *hr*. Then, the cytotoxicity effect of SB on cancer cells was investigated with MTT assay. The viability of HT-29 and HCT-116 cells was further decreased by higher doses of SB (6.25 to 200 *mM*). Our study revealed that SB could inhibit the proliferation of HT-29 (Figure 1A) and HCT-116 (Figure 1B) cell lines in a concentration and time-dependent manner.

### The IC_50_ calculated for SB

**Figure 2.**
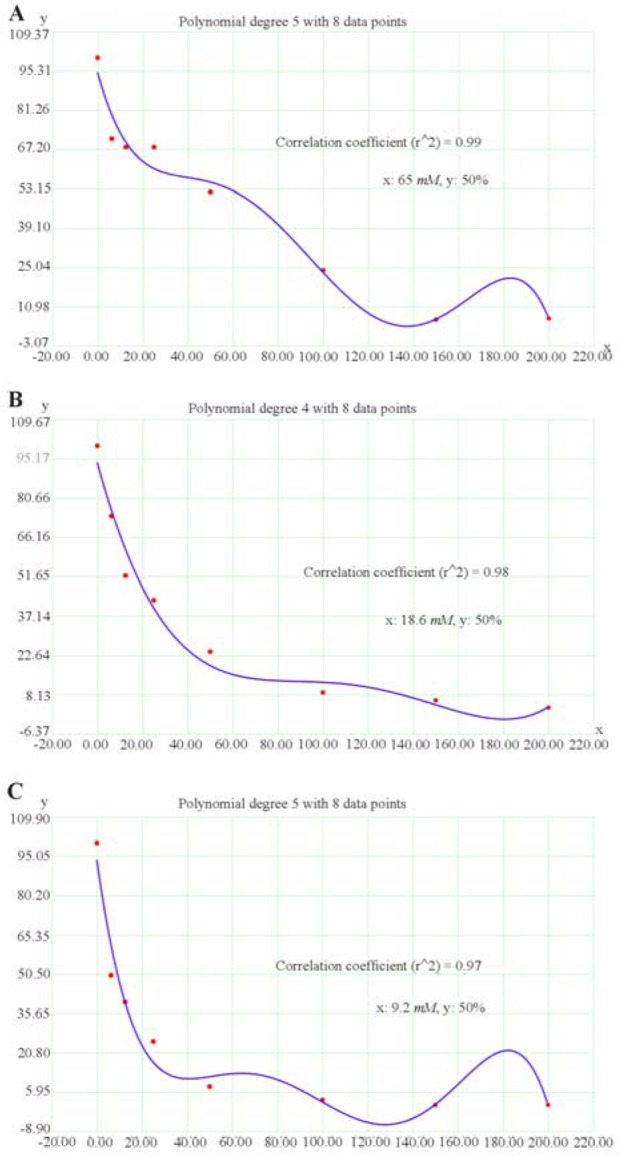
Regression analyses to calculate the 50% inhibiting concentration (IC_50_) values for effect of sodium butyrate (SB) on HT-29 human colorectal cell line. The horizontal axis (x) represents the concentration (*mM*) and the vertical axis (y) represents the percentage of the cell viability. A) The IC_50_ value was 65 *mM* for 24 *hr* after treatment, B) 18.6 *mM* for 48 *hr* after treatment, and C) 9.2 *mM* for 72 *hr* after treatment.

**Figure 3.**
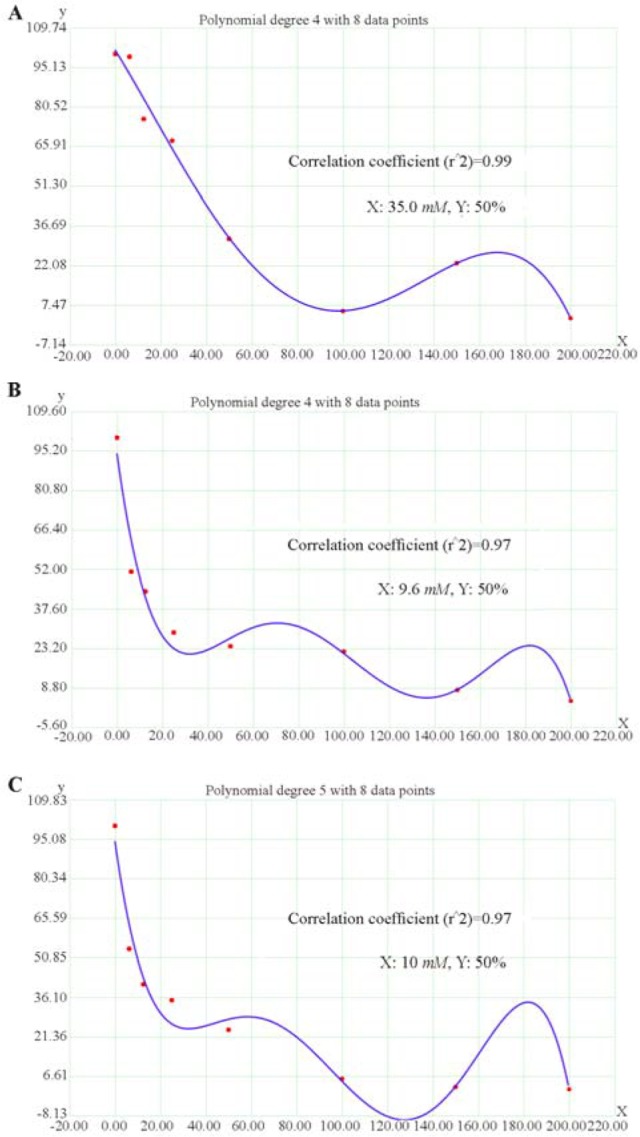
Regression analyses to calculate the 50% inhibiting concentration (IC_50_) values for effect of sodium butyrate (SB) in HCT-116 human colorectal cell line. The horizontal axis (x) represents the concentration (*mM*) and the vertical axis (y) represents the percentage of the cell viability. A) The IC_50_ value was 35.5 *mM* for 24 *hr* after treatment, B) 9.6 *mM* for 48 *hr* after treatment, and C) 10 *mM* for 72 *hr* after treatment.

The effective concentration of SB for the determination of the half-maximal inhibitory concentration (IC_50_) value was obtained by regression analyses of concentration-inhibition curves. The IC_50_ value for HT-29 human colorectal cell line was achieved as 65 *mM* for the 24 *hr* of SB treatment, 18.6 *mM* for 48 *hr* of SB treatment, and 9.2 *mM* for 72 *hr* of SB treatment (Figure 2). As well, the IC_50_ value for HCT-116 human colorectal cell line was 35.5 *mM* for 24 *hr* of SB treatment, 9.6 *mM* for 48 *hr* of SB treatment, and 10 *mM* for 72 *hr* of SB treatment (Figure 3). The IC50 of SB in HT-29 and HCT-116 human colorectal cancer cell lines was significantly decreased in 24, 48, and 72 *hr* in a time-dependent manner.

### Quantitative real-time PCR

#### HT-29 cell line:

**Figure 4.**
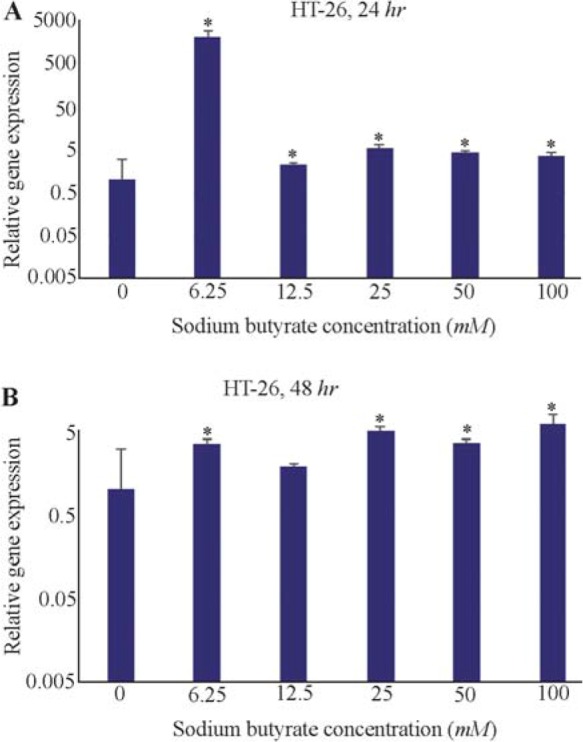
The effect of sodium butyrate (SB) on the *LHX1* mRNA expression in HT-29 cell line. A) Cells were cultured for 24 *hr* with 6.25 to 100 *mM* concentrations of SB at 37*°C*. B) Cells were cultured for 48 *hr* with 6.25 to 100 *mM* concentrations of SB at 37*°C*. *LHX1* mRNA expression was investigated using qRT-PCR. *GAPDH* was used as the internal control. *LHX1* mRNA expression increased in treated cells compared to control (0 *mM*). * Indicates a significant increase (p<0.05) *vs*. controls. All experiments were performed in duplicate.

The effect of SB was examined on *LHX1* mRNA expression in HT-29 human colorectal cancer cell line *in vitro* by incubating the cells in 6.25, 12.5, 25, 50, and 100 *mM* concentrations of SB for 24 and 48 *hr*. The concentrations of 150 and 200 *mM* were found to be toxic. After 24 *hr* of incubation with 6.25 to 100 *mM* concentrations of SB, *LHX1* mRNA expression significantly increased in all concentrations, compared with untreated cells as a control group (p<0.05) (Figure 3A); however, in higher concentration of SB, this fold change decreased in comparison with 6.25 *mM* concentration. This is probably owing to the very low numbers of cells at higher concentrations of SB treatment causing a denominator effect. The increased SB concentrations in the treatment were found to result in reduced cell numbers and enhanced cell death. After 48 *hr* of incubation, *LHX1* mRNA expression was significantly enhanced at concentrations of 6.25, 25, 50, and 100 *mM* SB, compared with untreated cells as a control group (p<0.05). Nonetheless, there was no significant increase in the concentration of 12.5 *mM* (p>0.05) (Figure 4).

### HCT-116 cell line

**Figure 5.**
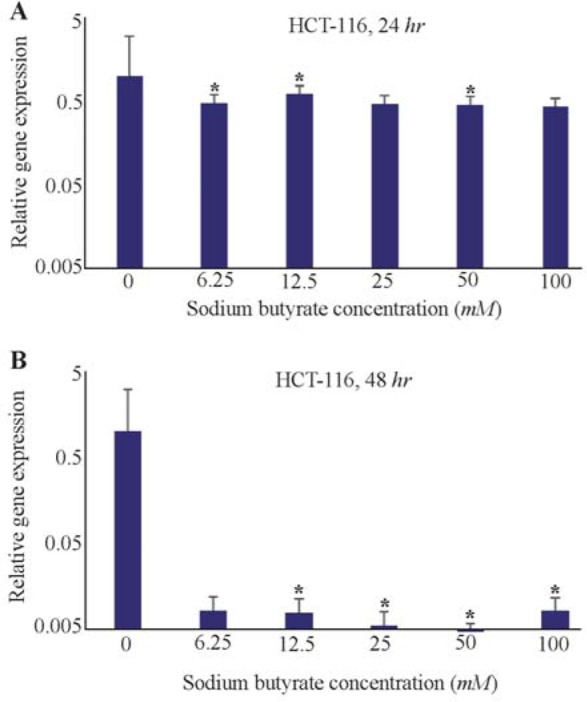
The Effect of sodium butyrate (SB) on *LHX1* mRNA expression in HCT-116 cell line. A) Cells were cultured for 24 *hr* with 6.25 *mM* to 100 *mM* of SB at 37*°C*. B) Cells were cultured for 48 *hr* with 6.25 *mM* 100 *mM* of SB at 37*°C*. *LHX1* mRNA expression investigated using qRT-PCR. *GAPDH* was used as an internal control. *LHX1* mRNA expression decreased in treated cells compared to control (0 *mM*). * Indicates a significant reduction (p<0.05) *vs*. controls. All experiments were performed in duplicate.

Also, the effects of SB on *LHX1* mRNA expression in HCT-116 human colorectal cancer cell line were investigated *in vitro* by incubating the cells in 6.25, 12.5, 25, 50, and 100 *mM* concentrations of SB for 24 and 48 *hr*. 24 *hr* after treatment with SB, *LHX1* mRNA expression significantly decreased at concentrations of 6.25, 12.5, 50, and 100 *mM* SB, compared with untreated cells as a control group (p<0.05). However, there was no significant decrease at the concentration of 25 *mM* (p>0.05) (Figure 5A). Likewise, 48 *hr* after treatment with SB, *LHX1* mRNA expression was significantly down-regulated in all concentrations of 6.25 to 100 *mM* SB, compared with untreated cells as a control group (p<0.05) (Figure 5B).

## Discussion

Acetylation is a chief part of the gene expression regulation 31,32 and is controlled by the opposite function of the HAT and HDAC enzymes 7. The dysregulated expression of HDAC enzymes is often seen in cancers 31,32. HDAC can regulate the expression of a large number of genes by direct interaction with transcription factors such as P53, E2f, Stat3, NF-KB, retinoblastoma protein, and TFIIE 11 affecting angiogenesis, cell cycle arrest, apoptosis, and the differentiation of different cell types 33,34. LHX1 is one of the transcription factors involved in the transcription of *HDAC8* gene 12. Despite the normal expression of *HDAC8* in healthy organs, its expression in tumor tissues is up-regulated 35,36. The selective pharmacological inhibition of HDACi represents a novel treatment for cancer therapy 18,33,34, 37,38. One of the HDACi is SB 22,23. In 2010, Ooi *et al* examined the effects of SB and their analogs in HT-29 cancer cells and observed that the 5 *mmol/L* concentration of SB resulted in decreased proliferation, increased apoptosis, and the reduction of HDAC activity 39. The findings of the presents study are consistent with the above information. The cytotoxicity of SB in HT-29 and HCT-116 human colorectal cancer cell lines was examined by using MTT assay. Our results revealed that SB could inhibit the proliferation of both HT-29 and HCT-116 cell lines in a concentration- and time-dependent manner. In HT-29 cell line, the viability of cells decreased to 52, 52, and 50% after 24, 48, and 72 *hr* of treatment, respectively. Besides, in HCT-116 cell line, the cell viability was diminished to 68, 51 and 54% after 24, 48, and 72 *hr* of treatment, respectively.

In the present study, for the first time, the effect of SB on the *LHX1* mRNA expression was investigated. In 2009, Haberland *et al* examined the relationship between *HDAC8* and homeobox transcription factors of *LHX1* and *Otx2* using PCR techniques in mice and concluded that the inappropriate expression of these transcription factors suppressed HDAC8 40. In 2011, Dormoy *et al* reviewed the transcription factor of LHX1 as a new oncogene in kidney cancer cells. They showed *LHX1* gene was re-expressed in kidney cancer and it is expressed in large quantities in kidney cancer cells, whereas in the normal kidney cells, it appears with a low expression level. On the other hand, they identified that the reduction of *LHX1* expression can lead to an increase in apoptosis and a decrease in cell proliferation after 72 *hr* 41. In addition, Saha *et al* have assessed the effects of an HDAC8 inhibitor on the transcription factors of Otx2 and LHX1 in mice. Their results depicted that HDAC8 suppresses the inappropriate expression of Otx2 and LHX1 and these two transcription factors are adjusted by HDAC8 42. Also, according to the literature, it was found that butyrate is able to stop cell cycle, differentiation, and apoptosis in a number of cell lines by inhibiting HDAC 43–45. SB affects the expression of genes by binding to the transcription factors. In this study, the effect of SB on *LHX1* as a transcription factor of HDAC8 in colorectal cancer cell lines was investigated. Existing documents have shown the inappropriate expression of *LHX1* in cancers that leads to the increased transcription, growth, and proliferation, as well as inhibition of cancer cell apoptosis 17,41. In the current study, it was expected that SB would act as a drug reducing the expression of *LHX1*. Our findings showed that treatments with SB significantly decreased the expression of *LHX1* in HCT-116 cells in comparison with untreated cells (p<0.05). However, to our surprise, the expression of *LHX1* significantly increased in HT-29 cell line, compared with untreated cells. Our results are well in line with that of Rocha *et al* that observed different effects of SB, as HDACi, on the expression of Estrogen Receptor (ERα). They expected that SB would lead to an increase in the ERα expression, while the opposite was found and the ERα expression was reduced 46. They suggested that treatment duration time and used concentrations may be critical in these effects 46. According to our results, Wang *et al* showed that HDACi could, *via* HDAC8/YY1, cause suppression of mutant P53 in breast cancer. HDAC8 reacts with YY1 transcription factor and adjusts the transcriptional activity. They figured out that treatment with SAHA and SB can inhibit the HDAC8 and YY1 association, enhance the YY1 acetylation, and eventually suppress the YY1-induced transcription of p53. They, also, determined that the network of HDAC8 and YY1 prevents the proliferation of breast cancer cells 47.

## Conclusion

The current study indicated that SB had anticancer activities and inhibits the growth of HT-29 and HCT-116 human colorectal cancer cell lines. Moreover, the results of this study showed that *LHX1* mRNA expression level was significantly different between two human colorectal cancer cell lines (HT-29 and HCT-116) due to SB treatment. In HT-29 human colorectal cell line, the significant increase of *LHX1* mRNA expression was observed after 24 and 48 *hr* of incubation time. On the contrary, SB led to a significantly down-regulated *LHX1* expression level at 24 and 48 *hr* of incubation time in HCT-116 human colorectal cell line. Altogether, these results indicated that there is no similar effect of SB on these different cell lines. Worthy of note, the histopathology origins of the used human colorectal cell lines in this study are distinguished. HT-29 is a cell line with adenocarcinoma origin derived from colon ascendens and colon with Dukes’ C stage (Involvement of lymph nodes) 48,49. HCT-116, on the other hand, has a carcinoma tissue origin and is derived from colon ascendens with Dukes’ D stage (Widespread metastases) 50–52. Moreover, the molecular features of these colon cancer cell lines are different 53; thus, their response to drugs is supposed to be distinct. SB might be capable of both repressing and inducing the expression of different genes. In this study, the expression of *LHX1* gene was investigated in untreated and treated colorectal cells and different effects of SB on *LHX1* mRNA expression were revealed in two different human colorectal cancer cell lines. Future studies are needed to evaluate the effect of SB on *LHX1* mRNA expression in other human colorectal cancer cell lines as well as other cancer cell lines.
